# What Is in the Field for Genetics and Epigenetics of Diabetic Neuropathy: The Role of MicroRNAs

**DOI:** 10.1155/2021/5593608

**Published:** 2021-10-06

**Authors:** V. Spallone, C. Ciccacci, A. Latini, P. Borgiani

**Affiliations:** ^1^Department of Systems Medicine, Endocrinology Section, University of Rome Tor Vergata, Rome, Italy; ^2^UniCamillus, Saint Camillus International University of Health Sciences, Rome, Italy; ^3^Department of Biomedicine and Prevention, Genetics Section, University of Rome Tor Vergata, Rome, Italy

## Abstract

Despite the high prevalence of diabetic neuropathy, its early start, and its impact on quality of life and mortality, unresolved clinical issues persist in the field regarding its screening implementation, the understanding of its mechanisms, and the search for valid biomarkers, as well as disease-modifying treatment. Genetics may address these needs by providing genetic biomarkers of susceptibility, giving insights into pathogenesis, and shedding light on how to select possible responders to treatment. After a brief summary of recent studies on the genetics of diabetic neuropathy, the current review focused mainly on microRNAs (miRNAs), including the authors' results in this field. It summarized the findings of animal and human studies that associate miRNAs with diabetic neuropathy and explored the possible pathogenetic meanings of these associations, in particular regarding miR-128a, miR-155a, and miR-499a, as well as their application for diabetic neuropathy screening. Moreover, from a genetic perspective, it examined new findings of polymorphisms of miRNA genes in diabetic neuropathy. It considered in more depth the pathogenetic implications for diabetic neuropathy of the polymorphism of MIR499A and the related changes in the downstream action of miR-499a, showing how epigenetic and genetic studies may provide insight into pathogenetic mechanisms like mitochondrial dysfunction. Finally, the concept and the data of genotype-phenotype association for polymorphism of miRNA genes were described. In conclusion, although at a very preliminary stage, the findings linking the genetics and epigenetics of miRNAs might contribute to the identification of exploratory risk biomarkers, a comprehensive definition of susceptibility to specific pathogenetic mechanisms, and the development of mechanism-based treatment of diabetic neuropathy, thus addressing the goals of genetic studies.

## 1. Introduction: Unresolved Clinical Issues in Diabetic Neuropathy

Both distal symmetric polyneuropathy (DPN) and cardiovascular autonomic neuropathy (CAN) are common complications of diabetes. DPN and CAN affect 30% and 20%, respectively, of the unselected population with diabetes, and their prevalence increases according to age and diabetes duration [1, 2]. Moreover, they both start early in the natural history of diabetes and are present also in prediabetes with a prevalence from 5.7% to 13% for DPN and 11% for CAN [3, 4]. Beyond the impact on the quality of life of painful forms of DPN and the clinical forms of autonomic neuropathy and the primary pathogenetic role of DPN in foot complications, both DPN and CAN have a heavy toll on survival also before the development of foot ulceration with an increased (up to 4 times) risk for mortality [5–9]. Notwithstanding, diabetic neuropathy is without doubt the least screened and diagnosed complication [10].

Moreover, not all patients needing symptomatic relief receive appropriate treatment, the latter is not universally effective [11, 12], and conclusive evidence-based efficacy of disease-modifying treatments is still lacking, or limited evidence for some of them has not yet reached the necessary requirements of the main regulatory agencies for their inclusion in guidelines. Thus, there are a number of unresolved clinical issues in the field of diabetic neuropathy, such as screening implementation, understanding of the mechanisms, identification and qualification of valid biomarkers, and developing disease-modifying treatments.

## 2. How May Genetics Address Diabetic Neuropathy Needs?

Even when considering the most effective preventive strategy (i.e., intensive glycemic control in type 1 diabetes that is able to prevent up to more than 60% of new cases of CAN and DPN), it is a matter of fact that for many patients (2% yearly), current strategies for optimizing glucose control are insufficient to fully prevent or delay the development of neuropathic complications [13]. Thus, the development and progression of neuropathic complications in any single patient cannot be completely anticipated by the control of hyperglycemia or other risk factors, and, consequently, genetic factors come into play. Indeed, the wide interindividual variability observed in diabetic neuropathy, in terms of susceptibility, clinical manifestations, and disease severity, has suggested that also genetic factors may influence the natural course of development of these complications.

The search for biomarkers, i.e., a measurable indicator of a pathophysiological condition, has become a main research topic in the field of DPN with the need to identify outcome biomarkers for clinical efficacy in clinical trials (surrogate for clinical endpoint), sensitive biomarkers for the very early stages of disease (useful to discern which patients in a preclinical stage are at a higher risk of developing clinical DPN), prognostic biomarkers for the tough consequences of DPN, and biomarkers pertaining to pathogenetic mechanisms in order to identify responsive patients to therapeutic agents targeted at these mechanisms [14, 15].

Genetics may address diabetic neuropathy needs by providing exploratory genetic biomarkers of susceptibility for disease development, by giving insights into the pathogenesis of neuropathy and neuropathic pain, and by shedding light on how to select the responders to treatment.

## 3. Candidate Gene and GWAS Approach

Genetics has in turn its own needs and requirements. First of all, two main different approaches are possible. The candidate gene approach starts with a hypothesis based on current knowledge of what kind of gene you intend to look for. It involves a limited number of variants, selected in candidate genes, and requires lower statistical power. Regarding diabetic neuropathy, candidate genes should encode proteins involved in the known mechanisms of nerve protection or damage in diabetes.

Genome-wide association study (GWAS) involves scanning the entire genome (or codifying genes) from different people, using platforms investigating thousands of variants [single-nucleotide polymorphisms (SNPs)] and looking for consistent difference between frequencies in variants associated with a disease. Without a priori-hypothesis, newly identified genetic markers can open to new knowledge. GWASs require large sample sizes (large-scale biobanks), rigorous thresholds for statistical significance (*p* < 5 × 10^−8^), wide coordination between different researchers and centers (Consortia), and considerable resources and replication studies [16].

Both these approaches are aimed at identifying genes and polymorphisms associated with the disease. They will then require further investigations to understand their possible functional role in the activity of the encoded proteins. Thus, the applicability of genetic variability studies for diabetic neuropathy involves the identification of genetic variants capable of influencing the level or the function of a clinical variable of interest for the complication, leading to pathogenetic consequences. Moreover, further studies should then verify if these genetic variants are associated with particular manifestations through a genotype-phenotype study.

## 4. Genetics of DPN: Where We Are

While genetic research has provided wide data on genetic variants associated with the risk of diabetic nephropathy and diabetic retinopathy, relatively little has been done regarding the genetics of neuropathy [17, 18] as also documented by less than half the number of publications than those for retinopathy and less than a third of those for nephropathy.

As expected, research on the genetics of diabetic neuropathy has mainly been oriented towards its pathogenesis. These multiple and interconnected mechanisms include abnormalities in glucose or lipid metabolism, oxidative stress, inflammation, endothelial dysfunction, endoplasmic reticulum stress, impaired nerve function, gene expression, and DNA damage [19]. These pathways might be at various stages the target of proteins encoded by genes whose polymorphisms are documented as relevant in diabetic neuropathy.

Four of these genes [i.e., the ACE gene, methylenetetrahydrofolate reductase (MTHFR) gene, glutathione peroxidase-1 (GPx-1) gene, and catalase (CAT) gene] have received greater attention, and even meta-analyses are available, with their polymorphisms possibly involved in the increased renin–angiotensin system activity, hyperhomocysteinemia, and reduced defense against oxidative stress [20]. Moreover, the pentose phosphate pathway has been considered for its pathogenetic relevance in hyperglycemic state, a potential therapeutic approach, and genetic variability, which has been documented for genes of thiamine transporter [21], transketolase, and glyoxalase [22, 23]. In addition, the role of genetic factors in the development of neuropathic pain has become increasingly recognized, with documentation in patients with painful DPN of variants of genes of *μ*-opioid receptor [24], a purinergic receptor [25], and the sodium channel Nav1.7 [26–28], possibly responsible for changes in opioid pain modulation or in increased excitability.

Finally, four GWASs are available in diabetic neuropathy, 3 from the datasets from UK Genetics of Diabetes Audit and Research in Tayside Scotland (GoDARTS) and pertinent to the phenotype of neuropathic pain and foot ulceration [29–31] and the most recent developed in the US Action to Control Cardiovascular Risk in Diabetes (ACCORD) study [32].

Thus, there is increasing attention given to the genetics of diabetic neuropathy, and although a suitable genetic biomarker has not yet been developed, promising findings can be seen on the horizon.

This review is focused on the genetics and epigenetics of microRNAs in diabetic neuropathy, given the research done by our group on this topic in the last few years.

## 5. The Case of MicroRNAs for Diabetic Neuropathy

MicroRNAs (miRs or miRNAs) are small RNA molecules (20–22 nucleotide length) that act as regulators in biological processes. At least 20–30% of all human genes are regulated by miRNAs through targeting sequences in their 3′ untranslated region. DNA regulatory regions might be involved in the control of development processes, hematopoietic cell differentiation, apoptosis, cell proliferation, and organ growth and in disease development. Increasing evidence supports the involvement of miRNAs in diabetes and its micro- and macrovascular complications [33].

### 5.1. miRNA Expression and Diabetic Neuropathy in Animal Models

Regarding diabetic neuropathy, in the last decade, very few studies have addressed miRNA expression in diabetic neuropathy with it being almost exclusively preclinical [34, 35]. Studies in animal models of diabetes and diabetic neuropathy have documented changes in the expression of a few miRNAs in peripheral nerve structures as related to changes in key points of pathways known as or presumed to be involved in the diabetes-related pathogenesis of nerve damage, regeneration, and in pain generation [36–43] (Table 1). In some studies, miRNAs target novel possibly pathogenetic mechanisms and miRNA mimics or anti-miR is utilized to confirm the hypothesized biological effect. This is the case of miR-25, found to be reduced in the sciatic nerve of diabetic mice and shown to act as a protective factor against advanced glycation endproducts and its receptor (AGEs-RAGE), as well as against oxidative stress through a reduction in protein kinase C alpha (PKC-alpha) and nicotinamide adenine dinucleotide phosphate (NADP) [42] or miR-146, which is reduced in the sciatic nerves of diabetic mice and rats and is negatively related to inflammatory cytokines and whose mimics produce beneficial structural and functional effects [40, 41] (Table 1). The protective effect of nanoparticle–miR-146a-5p polyplexes (nano-miR-146a-5p) was explored in DPN rats. Nano-miR-146a-5p increased nerve conduction velocity and decreased nerve damage and demyelination, together with a decrease in inflammatory cytokines and an increase in myelin basic protein, implying that the protective effect on peripheral nerves was mediated through the regulation of the inflammatory response and apoptosis, leading to the suggestion of a regulation action of miR-146a-5p in the nuclear factor kappa-light-chain-enhancer of activated B cell (NF-*κ*B) signaling pathway [44].

### 5.2. miRNA Expression and Diabetic Neuropathy in Humans

Very few studies have explored the association between the expression of miRNAs and diabetic neuropathy in humans. In a study hampered by an unclear definition of both type 2 diabetes and diabetic neuropathy, miR-199a-3p was found upregulated in the plasma of 60 patients with type 2 diabetes and in the lower limb skin of 30 patients with type 2 diabetes and DPN, compared to samples of 5 and 20 healthy volunteers, respectively [45]. Additionally, miR-199a-3p was shown in vitro to downregulate the serine protease inhibitor E2 (SerpinE2) that is known to upregulate the tissue plasminogen activator (tPA) provided with thrombolytic activity. Upregulation of miR-199a-3p was suggested by the authors to exert a procoagulant action in skin peripheral circulation and thus involved in DPN pathogenesis [45] (Table 1).

In a collaborative study between diabetologists and geneticists, we evaluated in 49 patients with type 2 diabetes the expression of 6 candidate miRNAs and assessed the presence of (1) DPN using validated scoring systems for neuropathic symptoms and signs and quantitative sensory testing for vibratory and thermal perception thresholds and (2) CAN by four cardiovascular reflex tests (CARTs) [46]. We defined the presence of probable DPN based on 2 abnormalities among symptoms, signs, and vibratory or thermal perception thresholds and of early CAN with at least one abnormal CART [2, 47]. Patients with DPN, compared to those without, showed a higher expression of miR-128a (*p* = 0.015) and a lower expression of both miR-155 (*p* = 0.04) and miR-499a (*p* = 0.05), whereas patients with CAN, compared to those without, displayed only a lower expression of miR-155 (*p* = 0.05) [46].

Using ROC analysis, we found fair diagnostic accuracy for DPN and diabetic neuropathy (DPN and/or CAN) with both a model including all three miRNAs and a model with miR-128a plus miR-155. For diabetic neuropathy, the area under the ROC curve (AUC) was 0.817 and 0.801 for the three-miRNAs and two-miRNA model, respectively, the sensitivity was 75.9% and 80.6%, and specificity was 76.5% and 70.6%, respectively. For DPN, AUC was 0.815 and 0.802, sensitivity was 74.1% and 80.6%, and specificity was 76.2% and 70.6% for the three-miRNAs and two-miRNA model [46]. Thus, the combination of two or three of these miRNAs had diagnostic accuracy for diabetic neuropathy identification, supporting the potential use of these miRNAs as epigenetic biomarkers for diabetic neuropathy.

### 5.3. Functions of miR-128a, miR-155, and miR-499a

To understand the possible meaning of these associations, one might consider that in animal studies, miR-128a downregulates insulin signaling pathways [48], impedes adipogenesis, and promotes lipolysis [49]. An overexpression of miR-128a might exert a pathogenetic role in diabetic neuropathy through these heightened adverse metabolic effects. Both insulin resistance in the dorsal root ganglion and dysregulation of adipogenesis and lipid metabolism have been observed in animal models of type 2 diabetes and DPN and proposed as additional factors in the pathogenesis of diabetic neuropathy [50, 51].

On the other hand, miR-155 is a multifunctional miRNA: in animal studies, it enhances insulin sensitivity, regulates inflammation and immunity [52], and exerts a neuroprotective effect [53], as well as being downexpressed in white blood cells of subjects with nondiabetic peripheral neuropathies [54], thus supporting a role in inflammation and neural function. An association between biochemical inflammatory markers and both DPN and CAN has been shown in patients with type 2 diabetes [55–57], and chronic low-grade inflammation is considered a critical pathway in the pathogenesis of diabetic neuropathy [58]. Thus, miR-155 expression changes might contribute to the inflammatory and immune-mediated pathogenetic mechanisms of diabetic neuropathy.

Finally, miR-499a, preferentially expressed in heart, skeletal muscle, and areas of the central autonomic network (nucleus ambiguous), has been found in animal models to be a regulator of the apoptotic pathway and mitochondrial fission in cardiomyocytes in response to ischemia or mechanical stress [59, 60] and was proposed as a marker of acute myocardial infarction and its severity [61, 62]. In fact, miR-499a targets the gene of phosphatase calcineurin A (CnA) that dephosphorylates the GTPase dynamin-related protein 1 (Drp1), the major mitochondrial fission protein. Dephosphorylated Drp1 migrates from the cytosol to the mitochondrial outer membrane and promotes mitochondrial fission and cell apoptosis [59, 60]. miR-499a overexpression might inhibit this pathway and prevent mitochondrial fission and apoptosis. Mitochondrial dynamics, i.e., fusion and fission, is also relevant in neuronal function, and mitochondrial dysfunction is believed to play a role in neurodegenerative diseases [63, 64] as well as in diabetic neuropathy. In murine models of diabetic neuropathy, Drp1 seems to mediate the hyperglycemia-driven mitochondrial damage in sensory neurons, and overactive mitochondrial fission in DRG neurons is suggested as a pathogenetic mechanism of diabetic neuropathy [65]. In addition, an imbalance in mitochondrial fusion and fission involving Drp1 was also shown as being responsible for diabetes-induced deficits in synaptic plasticity observed in the hippocampus in animal models of diabetes [66]. Thus, the role of miR-499a in neurological diabetic complications would appear to be fully supported.

## 6. A Genetic Perspective: Polymorphisms of *MIR146A*, *MIR128A*, and *MIR27A* Genes

Although the majority of studies are focused on miRNA expression profile investigation, recent studies have shown that also polymorphisms in miRNA genes may alter a wide spectrum of biological mechanisms and could play a role in the susceptibility to several human diseases, including diabetic neuropathy. In this regard, in our earlier studies, we used a genomic approach to investigate the relationship between miRNAs and diabetic neuropathy. The first study explored genetic polymorphisms in miRNA regions in relation to the susceptibility to type 2 diabetes [67]. This study assessed thirteen miRNAs as candidate loci—selected according to literature data and to a computational analysis—in 163 Italian subjects with type 2 diabetes and 185 healthy controls and found 6 newly described variants in addition to 9 SNPs already present in databases. In a case-control association study, two polymorphisms were found associated with type 2 diabetes susceptibility, i.e., the G allele of rs895819 in MIR27A with a protective effect (odds ratio = 0.58 and *p* = 0.008) and the G allele of rs531564 in MIR124A as a risk allele (odds ratio = 2.15, *p* = 0.008). This was the first report of genetic polymorphisms in miRNA regions as possible contributors to type 2 diabetes susceptibility [67].

Subsequently, we evaluated the possible contribution of genetic polymorphisms of miRNA genes in susceptibility to DPN and CAN [68]. Nine polymorphisms were studied in a sample of 132 patients well-defined for the diagnosis of probable DPN (based on the presence of both neuropathic symptoms and signs) and early or confirmed CAN (according to one or more abnormal CARTs, respectively). The study found an association of the rs2910164 (G>C) in MIR146A and rs11888095 (C>T) in MIR128A with DPN susceptibility. In particular, the C allele of rs2910164 in MIR146A was seen to be a protective variant (odds ratio = 0.46, *p* = 0.032), while the variant T allele of rs11888095 in MIR128A was associated with a high risk of developing DPN (odds ratio = 2.01, *p* = 0.007). The latter association was also confirmed after correction for BMI, age, disease duration, HbA1c, and gender (adjusted odds ratio = 4.89, *p* = 0.002). Moreover, the same SNP in MIR146A showed a protective effect for early CAN (adjusted odds ratio = 0.32, *p* = 0.052) and for confirmed CAN (adjusted odds ratio = 0.13, *p* = 0.041), while a polymorphism in MIR27A was associated with a higher risk of developing early CAN (adjusted odds ratio = 3.43, *p* = 0.023). An association of SNPs of MIR128A and MIR146A was also present in multiple linear regression analysis with the severity of DPN and CAN, namely, the scores for neuropathic symptoms/signs (*p* = 0.026 for MIR128A) and the score based on CARTs (*p* < 0.0001 for MIR146A) [68]. This represented the first observation of an involvement of genetic variability in miRNA genes in diabetic neuropathy susceptibility.

We described above that in animal models of diabetic neuropathy, the expression of miR-146a was found to be downregulated and inversely associated with levels of inflammatory cytokines [41] and, further, that miR-146a mimics had protective effects on peripheral nerves [40], possibly mediated by the inhibition of inflammatory response and apoptosis through the regulation of NF-*κ*B [44]. Similarly, we presented earlier metabolic effects of miR-128a and how these might provide a meaning to the association between its expression levels and diabetic neuropathy. It is more difficult to disentangle the value of the weak link of the polymorphism of MIR27A with CAN. In addition to the evidence of its role in tumor biology, some studies have suggested that miR-27a expression was upregulated in the T cells of patients with multiple sclerosis and that in murine T cells, it impaired regulatory T cell (Treg) generation by downregulating runt-related transcription factor 1 (RUNX1) and then the forkhead box P3 (Foxp3), i.e., the master transcription factor in maintaining differentiation and suppressive function of Tregs [69]. On the other hand, the involvement of the miR-27 family in metabolic disorders and in hepatic glucose metabolism was described, with it having forkhead box O1 (FOXO1) as a downstream target [70].

The polymorphisms in these miRNA genes might affect the expression or the downstream action of the corresponding miRNAs and lead in some way to changes in metabolic and inflammatory/immune mechanisms active in the scenario of diabetic neuropathy.

### 6.1. Polymorphisms of miRNA Genes: MIR499A

In a more recent study, in 150 participants with type 2 diabetes, we analysed the rs3746444 SNP in the MIR499A gene to evaluate its association with susceptibility to DPN and CAN [71]. We found that the GG genotype after correction for age, sex, BMI, and HbA1c was associated with the risk of early CAN (adjusted odds ratio = 16.08, *p* = 0.002), confirmed CAN (adjusted odds ratio = 35.02, *p* = 0.0005), and DPN (adjusted odds ratio = 6.56, *p* = 0.037). In addition, MIR499A GG genotype independently contributed to early CAN together with duration and HbA1c and to DPN together with duration, HbA1c, and age. Finally, the GG genotype was associated with worse values of neuropathic deficit score, i.e., Michigan Diabetic Neuropathy Score (*p* = 0.017), vibration perception threshold (*p* = 0.01), thermal thresholds (*p* = 0.01), and CART score (*p* < 0.001): in a multiple linear regression, the GG genotype was the main variable contributing to the CART score (*p* = 0.001). Thus, the rs3746444 GG genotype might represent a marker of higher risk of DPN and CAN and of CAN severity. We described above the hypothesized actions of miR-499a and its ability to prevent mitochondrial fission as well as the imbalance in mitochondrial fusion and fission as a potential pathogenetic mechanism of diabetic neuropathy [65].

### 6.2. mtDNA Copies and Diabetic Neuropathy

More insight into the meaning of the association between MIR499A polymorphism and diabetic neuropathy comes from a further study by our group [72] that measured in 125 patients with type 2 diabetes the number of mitochondrial DNA (mtDNA) copies and assessed DPN, CAN, and the polymorphism of MIR499A. The study found a decrease in the number of mtDNA copies in patients with type 2 diabetes compared to healthy controls (*p* = 2 × 10^−10^) and further differences between patients with and without DPN (*p* = 0.02) (not between those with and without CAN). In addition, the homozygous variant genotype for the rs3746444 polymorphism of MIR499A was associated with the number of mtDNA copies, particularly in patients with type 2 diabetes with values of 22.71 ± 8.65 in patients with AA+AG genotypes and 14.43 ± 5.29 in those with the GG genotype (*p* = 0.009).

Mitochondrial biogenesis is a defense modality against hyperglycemic load and hyperglycemia-driven oxidative stress in diabetes through the increase in the mass of the mitochondrial network, with mtDNA being a marker of this process [65]. Prolonged hyperglycemia and oxidative stress induce a decrease in the mtDNA copy number [73] as also found in dorsal root ganglia from mice with chronic diabetic neuropathy [74].

The study confirmed in people with type 2 diabetes the observations obtained in animal models of diabetic neuropathy and documented for the first time that the studied polymorphism in MIR499A might affect the number of mtDNA copies, thus impairing mitochondrial biogenesis.

### 6.3. MIR499 Polymorphism and miR-499 Expression in DPN: Hypothesis for a Pathogenetic Role in DPN

**Figure 1:**
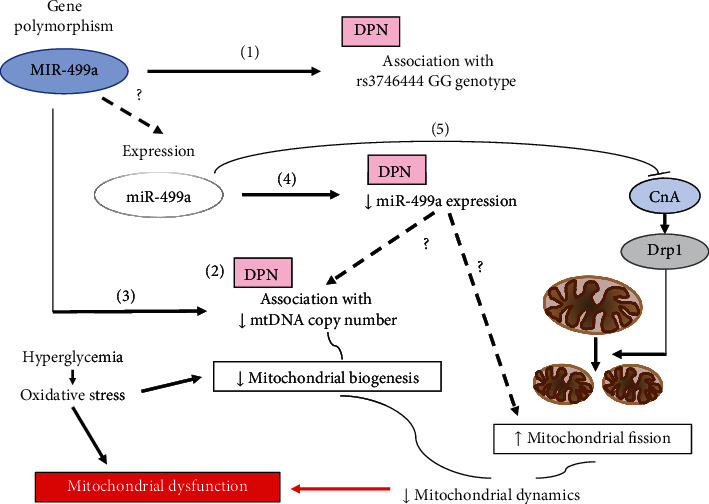
MIR499A polymorphism and miR-499a expression in DPN: hypothesis for a pathogenetic role in DPN. Mitochondrial dynamics are a continuous process of fusion, fission, biogenesis, and mitophagy that in neurons contrasts hyperglycemia-driven oxidative stress and maintains cellular bioenergetics [64, 73]. Mitochondrial dysfunction derives from persistent increase in metabolic load and oxidative stress in neurons in diabetes and is considered a relevant mechanism in the pathogenesis of DPN [65, 73]. Both dysregulation of the fission/fusion balance with increased fission and impaired biogenesis with reduced number of mtDNA have been found in diabetes. Recent studies have shown in people with type 2 diabetes (1) an association of the polymorphism rs3746444 of MIR499A with CAN and DPN [71], (2) a reduction in mtDNA copy number, more pronounced in the presence of DPN [72], (3) an association between this change in mtDNA copy number and the same polymorphism of MIR499A [72], (4) a reduced expression of miR-499a in subjects with DPN [46], and (5) in rat and human cardiomyocytes that miR-499a targets the gene of calcineurin (CnA), inhibits its expression and the CnA-mediated activation of dynamin-related protein (Drp) 1 responsible for mitochondrial fission and apoptosis [59, 60]. These findings allow the hypothesis that MIR499A polymorphism and changes in expression and function of miR-499a might affect both mitochondrial biogenesis and increase mitochondrial fission thus altering mitochondrial dynamics and leading to mitochondrial dysfunction and to DPN. It is not documented (dashed lines) that the studied MIR499A polymorphism affects miR-499 expression, and that in DPN, the reduced miR-499a expression is related to decreased mtDNA copy number and increased mitochondrial fission.

Thus, summarizing the findings regarding the miR-499a system in diabetic neuropathy, we have evidences of (1) an association of homozygous variant genotype for the rs3746444 polymorphism of MIR499A with CAN and DPN in type 2 diabetes [71], (2) a reduction in the mtDNA copy number in type 2 diabetes, which is more pronounced in the presence of DPN [72], (3) an association in these patients between this change in the mtDNA copy number and the same polymorphism of MIR499 [72], (4) a reduced expression level of miR-499a in subjects with DPN [46], and (5) the experimental observations in rat and human cardiomyocytes that miR-499 targets the CnA gene and inhibits its expression and the CnA-mediated activation of Drp1 responsible for mitochondrial fission and apoptosis [59, 60]. These data, taken together, allow for the hypothesis that the polymorphism of MIR499A and reduced miR-499a expression may dysregulate mitochondrial biogenesis as suggested by the reduced mtDNA number and increase mitochondrial fission thus impairing mitochondrial dynamics. Mitochondrial dynamics are a pillar of mitochondrial function in neurons, able to restore homeostasis, to contrast hyperglycemia driven oxidative stress, and to maintain optimal cellular bioenergetics [64, 73]. In this way, mitochondrial dysfunction can occur, which represents a newly recognized essential mechanism in the pathogenesis of DPN [65, 73] as well as of other neurological diseases [63, 64] (Figure 1).

## 7. Genotype-Phenotype Association for Polymorphisms of miRNA Genes

A question might arise: are the observed polymorphisms of miRNA genes able to change the corresponding miRNA expression? In the previously cited study [46], in 49 patients with type 2 diabetes with and without DPN and CAN, the relationship between the expression of 6 miRNAs and the genotypic classes of the corresponding miRNA genes was assessed. We found, after adjustment for age, sex, and diabetes duration, that the rs767649 variant allele in the *MIR155* promoter region was associated with a higher expression of this miRNA compared with the wild-type allele (adjusted *p* = 0.013). The rs767649 variant allele is localized in the promoter region of the MIR155 gene and might alter the binding of transcriptional factors, which are mainly inflammatory mediators and bacterial or viral-derived toll-like receptor ligands [75] in this way increasing miR-155 expression. In two previous studies, the rs767649 variant allele was associated with a reduced susceptibility to type 1 [75] and type 2 diabetes [76].

Moreover, the rs11888095 SNP polymorphism in MIR128A was associated with higher expression level of miR-128a (adjusted *p* = 0.022). The polymorphism of MIR128A had been previously associated in a larger group of patients with type 2 diabetes with a higher risk of developing DPN [68], thus the association, here documented for the first time, between the variant allele of MIR128A and a higher risk to develop DPN might be mediated by changes in miRNA expression.

In our study, we failed to observe an association of the polymorphism in the rs3746444 polymorphism of MIR499A and the miR-499a expression. It is possible that other mechanisms contribute to changing the expression of this miRNA or that the variant allele might be involved in changing the miRNA targets rather than in regulating its expression.

Despite the small size of the studied population, this genotype-phenotype association study has shown for at least two miRNA genes that the genetic polymorphism corresponds to changes in miRNA expression with a consistency between what observed for the variant alleles and expression in their association with diabetic neuropathy. These results link the genetics and epigenetics of miRNAs and support their potential usefulness as exploratory predictive biomarkers and therapeutic targets.

## 8. What Does MicroRNA Genetics Put in Place for the Challenge of Diabetic Neuropathy?

The genetics of diabetic neuropathy is starting to offer results towards the achievement of biomarkers of risk and pathogenetic mechanisms, to provide support to known hyperglycemia-related mechanisms and to suggest new pathogenetic pathways and to validate therapeutic targets.

Genetic information might be integrated into a multibiomarker approach including clinical, metabolic, and imaging phenotyping to define individual susceptibility to diabetic neuropathy and its therapeutic options.

Genetic research is challenging and requires a joint force strategy to improve sample size and phenotyping. It is worthwhile proceeding, and the results in some fields might be nearer to our goals than we suspect.

In this scenario, the genetics of microRNAs is at a very preliminary stage. Notwithstanding, the study of genetics and epigenetics of miRNAs may well contribute to the identification of exploratory biomarkers of risk and pathogenetic mechanisms of diabetic neuropathy and to a comprehensive definition of susceptibility to specific pathogenetic mechanisms to favor a tailored mechanism-based treatment or prevention. The data presented here would appear to anticipate a promising development.

## Figures and Tables

**Table 1:** Studies exploring the association between miRNAs and diabetic neuropathy.

miRNA	Target	Expression	Observation	Author, year
Animal studies				
miRNA-29b	Neurotrophic activity (↑)	Downregulated	In diabetic rats, miRNA-29b was downregulated in dorsal root ganglia neurons and associated with apoptosis and axonal swelling	Zhang, 2014 [36]
mmu-let-7i	NF-*κ*B neurotrophic activity (↑)	Downregulated	In DPN type 1 diabetes mice, mmu-let-71 was reduced and mmu-mir-341 increased in dorsal root ganglia neurons; let-7i miRNA mimics and mmu-miR-341 anti-miR improved structural and functional abnormalities	Cheng, 2015 [37]
mmu-miR-341	Neurotrophic activity? (↓)	Upregulated		
3pmiRNA-190a-5p	SLC17A6 (↓)	Downregulated	In DPN mice models, 3pmiRNA-190a-5p was downregulated and SLC17A6 overexpressed	Yang, 2017 [38]
miRNA-9	CALHM1 (↑)	Upregulated	In painful DPN rat model, miRNA-9 was overexpressed in spinal dorsal horn neurons and related to CALHM1	Liu, 2017 [39]
miRNA-146a	Proinflammatory genes (↓)	Downregulated	In diabetic mice, miR-146a mimics improved sciatic nerve vascular function, axonal myelination, and peripheral nerve function	Liu, 2017 [40]
miRNA-146a	NF-*κ*B and inflammatory cytokines (↓)	Downregulated	In diabetic rats with DPN, miR-146a was reduced in sciatic nerves and negatively related to TNF-*α*, IL-1*β*, and NF-*κ*B	Feng, 2018 [41]
miRNA-25	PKC-*α* and NADP (↓)	Downregulated	In diabetic mice, miRNA-25 was reduced in sciatic nerves and associated with increase in ROS. miR-25 mimics decrease NADP and PKC-a, and miRNA-25 anti-miR increases AGEs and RAGE	Zhang, 2018 [42]
miRNA-29c	PRKCI (↓)	Upregulated	In diabetic mice, miRNA-29c was increased in DRG and sciatic nerve and suppresses axonal growth by inhibiting PRKCI	Jia, 2018 [43]
miR-146a-5p	Inflammatory response and apoptosis	Downregulated	In diabetic rats with DPN, nano-miR-146a-5p had a protective effect on peripheral nerves (↑ NCV and ↓ nerve damage and demyelination) together with ↓ inflammatory cytokines and ↑ myelin basic protein	Luo, 2019 [44]
Human studies				
miRNA-199a-3p	SerpinE2 (↓)	Upregulated	In 60 patients with type 2 diabetes and DPN, miRNA-199a-3p was upregulated in plasma and skin with consequent downregulation of SerpinE2 (and tPA with procoagulant effect)	Li, 2017 [45]
miRNA-128a	Insulin signaling pathways (↓), adipogenesis (↓), and lipolysis (↑) (miRNA-128a)	Upregulated	In 49 T2DM, miRNA-128a was upregulated, while miRNA-155 and miRNA-499 were downregulated in plasma in those with DPN and miRNA-155 was downregulated in those with CAN	Ciccacci, 2020 [46]
miRNA-155	Insulin sensitivity (↑), inflammation, immunity, neuroprotection (↑) (miRNA-155)	Downregulated		
miRNA-499a	Apoptotic pathway and mitochondrial fission through CnA and Drp1 (miRNA-499)	Downregulated		

AGEs: advanced glycation endproducts; CALHM1: calcium homeostasis modulator 1; CnA: calcineurin; Drp1: dynamin-related protein 1; IL-1*β*: interleukin 1 beta; NADP: nicotinamide adenine dinucleotide phosphate; NF-*κ*B: nuclear factor kappa-light-chain-enhancer of activated B cells; NCV: nerve conduction velocity; PKC-*α*: protein kinase C alpha; PRKCI: protein kinase C iota type; RAGE: receptor for advanced glycation endproduct; ROS: reactive oxygen species; SerpinE2: serine protease inhibitor E2; SLC17A6: gene for vesicular glutamate transporter 2; T1DM: type 1 diabetes; TNF-*α*: tumor necrosis factor alpha; tPA: tissue plasminogen activator.
